# Editorial: Odor information processing and stress response

**DOI:** 10.3389/fnbeh.2023.1142186

**Published:** 2023-01-31

**Authors:** Mutsumi Matsukawa, Mitsuhiro Fukuda, Takaaki Sato

**Affiliations:** ^1^Division of Anatomical Science, Department of Functional Morphology, Nihon University School of Medicine, Tokyo, Japan; ^2^Department of Radiology, University of Pittsburgh, Pittsburgh, PA, United States; ^3^Biomedical Research Institute, National Institute of Advanced Industrial Science and Technology, Osaka, Japan

**Keywords:** olfactory bulb (OB), anterior and/or posterior piriform cortex, functional roles in the olfactory pathway, olfactory cortical connectivity, odor-mediated behaviors, odor-associated stress and/or fear, olfactory system for emotional behaviors

Humans and animals constantly scan and assess their surrounding environment, making decisions and acting appropriately by exploring or avoiding certain areas. Sensory organs acquire multiplexed, multimodal sensory inputs from proximal and/or distal objects and send them to the brain, where bottom-up and top-down parallel processing transforms them into salient or multiple innate and/or learned information for appropriate behavioral decisions.

Three interconnected odor sensory systems, along with hundreds of distinct receptors, process olfactory information in a diverse and sophisticated manner. With or without stress expression and emotional reactions, the numerous behaviors evoked by this odor information are primarily connected to survival and reproduction in their environment. To better grasp these olfactory information processing and behavioral research frontiers, this research subject aims to investigate and compile numerous findings on odor-induced brain activity, behavior, and stress manifestation. Specifically, we examine five reports on odor-induced stress reactions and behavioral phenotypes.

Regarding stress information of environmental and internal odor worlds, Mori and Sakano review the inferential neural circuits and mechanisms involved in the two separate nasal streams to olfactory processing. Specifically, retronasal/interoceptive information may be processed in the medial portion of the olfactory-related areas (purple line in Figure 1a), while the orthonasal/exteroceptive information may activate the lateral portion (blue line in Figure 1a). As it is assumed that the orthonasal/exteroceptive pathway corresponds to the exogenous stress response and the retronasal/interoceptive pathway to the endogenous stress response, respectively, it is expected that the differences in the processing of olfactory information between these two nasal streams will be analyzed in more detail in the future.

**Figure 1:**
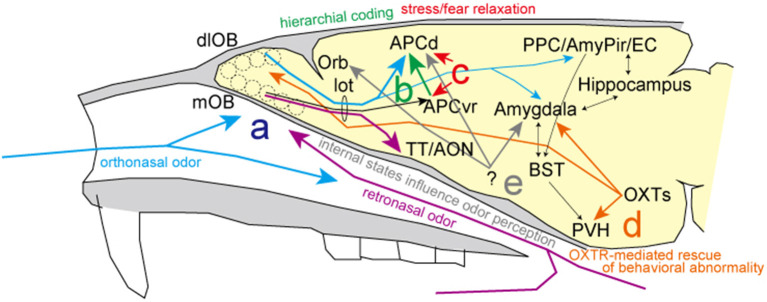
Summary of the research topic. **(a)** There are two putative streams of odor information processing: the blue line shows the orthonasal/exteroceptive stream in lateral olfactory areas and the purple line shows the retronasal/interoceptive stream in the medial olfactory areas. **(b)** Hierarchical coding for the fine discrimination of odors (green line). **(c)** Selective inhibition systems to alleviate odor-induced stress responses in the anterior piriform cortex (red lines). **(d)** Oxytocinergic modulatory neurotransmission for resolving odor-related behavioral abnormalities (orange lines). **(e)** Changes in fMRI signals are found in parallel with behavioral changes and depend on the internal state, particularly with hunger and satiety. AmPir, amygdalo-piriform transition area; AON, anterior olfactory nucleus; APCd, dorsal part of the anterior piriform cortex; APCvr, ventro-rostral part of the anterior piriform cortex; BST, bed nucleus of the stria terminalis; EC, entorhinal cortex; lot, lateral olfactory tract; dlOB, dorsolateral wall of the olfactory bulb; mOB, medial wall of the olfactory bulb; Orb, orbitofrontal cortex; OXTs, oxytocin neurons; PPC, posterior piriform cortex; PVH, paraventricular hypothalamic nucleus; TT, tenia tecta.

Diseases gradually alter the body odors of individuals. It is likely that animals instinctively avoid or discriminate individuals with such body odors to mitigate risk or stress. Sato et al. review excellent discrimination ability of mice for urinary odors of patients with prostate or bladder cancer, indicative of their different biomarker profiles. Similar to the four elemental R/G–Y/B color coding scheme in vision, it is hypothesized that elemental odor information is represented in the anterior piriform cortex by signal addition and subtraction driven by feedforward inhibitory signal in a hierarchical fashion (green line in Figure 1b). This hierarchical elemental odor coding is essential to enable fine odor discrimination between similar odors of various cancers or enantiomers. In addition, to make progress on creating cancer odor sensors that can identify diseases before traditional cancer markers in the plasma, much effort has been devoted to understanding the mechanism underlying fine odor discrimination.

Has the scent of Bulgarian roses or phytoncide ever relieved your stress? Matsukawa et al. discuss the significance of the anterior piriform cortex as an inhibitory regulatory system for odor-induced stress reactions and relaxation (red line in Figure 1c). Studies of brain areas involved in stress expression and its coping mechanisms reveal that the selective inhibitory system in the odor-induced stress response is not processed in the olfactory bulb but rather in the primary olfactory cortex, the piriform cortex. In addition to advancing our understanding of odor-induced and inhibitory processes of stress reactions, future clarification of the precise brain circuits and inhibitory systems involved will be valuable for treating stress disorders.

Osada et al. report an innovative investigation on the impact of coffee odor on rescue of behavioral impairments in mice with a heterozygous deficiency in oxytocin receptor (OXTR) expression. Repeated coffee odor delivery alters OXTR expression in some brain areas, including the amygdala and olfactory bulb, and can help to rescue abnormal behaviors along with other neurochemical systems (orange line in Figure 1d). Notably, a recent preprint reported that transcriptional lability of brain OXTR diversifies brain OXTR distributions and social behaviors (Zhang et al., 2022). As a system for addressing odor-induced behavioral disorders and as a regulatory system for brain processes, further advancements are anticipated.

As described by Mori and Sakano, hunger and thirst are the internal physiological stress and homeostatic challenges essential for driving food/water-searching and ingestion behaviors. Beyond this collection, we introduce an interesting review of human behavioral studies on how internal states, particularly hunger and satiety, influence odor perception (Shanahan and Kahnt, 2022). Hunger and satiety alter the way we perceive the scent of foods to be eaten and foods recently consumed. Odor sensitivity also seems to be affected: while sensitivity to food odors increases in the hungry state, it decreases after eating odor-matched foods. Behavioral changes are found in parallel with changes in fMRI signals in piriform cortex, amygdala, and orbitofrontal cortex (gray line in Figure 1e). Since multiple brain regions are involved in the odor-induced stress response, fMRI should be valuable for human and animal studies because it is both noninvasive and can provide a measure of whole-brain activity.

While these articles discuss stress, fear, and disease odors in relation to aversive scents, they also provide a variety of different features that show how olfaction is involved in different information–behavior control systems. It is believed that odor information can affect mood, behavior, and stress reactions in both humans and animals. In addition, human olfactory disorders have been associated with increased stress. Based on information from this Research Topic, interesting experiments employing fMRI or other novel strategies may shed new light on how olfaction and stress are connected.

## Author contributions

All authors listed have made a substantial, direct, and intellectual contribution to the work and approved it for publication.
